# Diurnal fluctuation in the number of hypocretin/orexin and histamine producing: Implication for understanding and treating neuronal loss

**DOI:** 10.1371/journal.pone.0178573

**Published:** 2017-06-01

**Authors:** Ronald McGregor, Ling Shan, Ming-Fung Wu, Jerome M. Siegel

**Affiliations:** 1 Department of Psychiatry and Biobehavioral Sciences, University of California at Los Angeles, Los Angeles, California, United States of America; 2 Brain Research Institute, University of California at Los Angeles, Los Angeles, California, United States of America; 3 Veterans Administration Greater Los Angeles Healthcare System, Neurobiology Research, North Hills, California, United States of America; Kent State University, UNITED STATES

## Abstract

The loss of specific neuronal phenotypes, as determined by immunohistochemistry, has become a powerful tool for identifying the nature and cause of neurological diseases. Here we show that the number of neurons identified and quantified using this method misses a substantial percentage of extant neurons in a phenotype specific manner. In mice, 24% more hypocretin/orexin (Hcrt) neurons are seen in the night compared to the day, and an additional 17% are seen after inhibiting microtubule polymerization with colchicine. We see no such difference between the number of MCH (melanin concentrating hormone) neurons in dark, light or colchicine conditions, despite MCH and Hcrt both being hypothalamic peptide transmitters. Although the size of Hcrt neurons did not differ between light and dark, the size of MCH neurons was increased by 15% in the light phase. The number of neurons containing histidine decarboxylase (HDC), the histamine synthesizing enzyme, was 34% greater in the dark than in the light, but, like Hcrt, cell size did not differ. We did not find a significant difference in the number or the size of neurons expressing choline acetyltransferase (ChAT), the acetylcholine synthesizing enzyme, in the horizontal diagonal band (HBD) during the dark and light conditions. As expected, colchicine treatment did not increase the number of these neurons. Understanding the function and dynamics of transmitter production within “non-visible” phenotypically defined cells has fundamental implications for our understanding of brain plasticity.

## Introduction

In mammals, waking and sleep states are generated and stabilized by the coordinated activity of different neuronal groups. Amongst them, hypocretin (Hcrt), histamine and acetylcholine producing neurons promote arousal whereas melanin concentrating hormone (MCH) neurons promote sleep. Consistent with their role in modulating each state, Hcrt, histamine and cholinergic neurons show the highest levels of activity during the active phase [1] [2] [3] [4] [5] [6] [7] whereas peak MCH release and unit activity has been reported to be prior to and during the inactive phase [8] [9] [10] [11]. In spite of the extensive evidence indicating circadian modulation of these systems, studies have so far assumed that the number of detectable neurons in each neurochemical group remains constant across the different behavioral states. Furthermore fluctuations in neuronal numbers have been generally associated with aging, pathological conditions, genetic manipulation, or specific experimental procedures. For example, in humans lesions to the posterior hypothalamus, where histaminergic neurons reside, generate hypersomniac phenotypes [12], whereas loss of hypocretin neurons results in narcolepsy [13] a sleep pathology characterized by excessive daytime sleepiness, as well as intermittent and uncontrollable episodes of falling asleep. Recent work showed that human narcolepsy was accompanied by an increase in the number of histamine cells [14] [15]. Loss of histamine in animals results in an inability to maintain waking in novel environments and slowing of the EEG in spite of normal amounts of sleep and wake [12], whereas loss of Hcrt cells produces narcoleptic phenotypes. Genetically modified animals can also express altered number of neurons compared to wild type controls. For example, narcoleptic dogs lacking the hypocretin receptor 2 have a higher number of cholinergic neurons in the pedunculopontine nuclei [16] [17], whereas the genetic rat model Flinders Sensitive Line displays a higher number of Hcrt neurons [18]. Castration of male mice [19] or lipopolysaccharide administration [20] decrease the number of Hcrt expressing neurons whereas an agonist of the estrogen receptor α decreases the number of MCH expressing neurons [21] and parasitic infection with *Trypanosoma brucei* reduces both Hcrt and MCH cell numbers [22], indicating that the cell number of these hypothalamic populations may be modulated in response to an environmental challenge.

Because of the circadian modulation of these systems and the evidence indicating that neuronal numbers can fluctuate in response to different challenges we sought to determine if there is a physiological variation in the number of neurons that express Hcrt, histamine and MCH.

## Materials and methods

### Subjects

Sixteen-week-old male mice (C57BL/6J) from Charles River Laboratories were used. Animals were kept in a room maintained at 22±1°C on a 12 h light (45 lux) dark (0.03 lux) cycle (lights on 7 AM and off 7 PM). All procedures were approved by the Institutional Animal Care and Use Committee of the University of California at Los Angeles and of the Veterans Administration Greater Los Angeles Health Care System.

### Light/Dark phase

Naïve mice (n = 16) were maintained undisturbed in their home cage in an isolated room, except for a weekly bedding change and food/water replenishment to ensure *ad libitum* feeding conditions. Starting seven days before sacrifice, the animals where kept completely undisturbed. In this experimental group the subjects were sacrificed at ZT 5 (5 hours after light onset) and at ZT 17 (5 hours after light offset).

### Colchicine administration

Twenty-six animals were subjected to intracerebroventricular (ICV) injection of either saline solution (n = 13) or saline solution containing 20 μg/μl of colchicine (n = 13). Anesthesia was induced with a mixture of ketamine/xylazine (100 mg/kg/15 mg/kg, i.p.) and then maintained with a gas mixture of isoflurane in oxygen (0.6–1.2%) after the animals were placed in the stereotaxic device. Body temperature was maintained with a water-circulated heating pad (Gaymar Industries, Orchard Park, NY, USA). The head was positioned in a stereotaxic frame and the skull was exposed. A single hole was drilled at coordinates corresponding to the lateral ventricle (AP: -0.5 mm, L: -1 mm, relative to bregma). A Hamilton microsyringe was lowered until the ventricle was reached (H: -2.8 mm, relative to the skull surface). Infusion was made in increments of 0.2 μl every 10 minutes for 40 minutes to obtain a final volume of 1 μl. The needle was held in place for another 10 minutes before slowly withdrawing it. Ventricular localization was confirmed by observing free flowing cerebrospinal fluid after the withdrawal of the needle. A small piece of sterile bone wax was placed over the hole and the skin sutured. All subjects recovered from the anesthesia within 30 minutes after the end of the procedure. All animals were carefully monitored and sacrificed 52 h later between ZT 13 and ZT 15 for immunohistochemical procedures and mRNA quantification.

### Tissue processing

For tissue collection, all animals were deeply anesthetized with pentobarbital (Nembutal, 100 mg/kg, i.p.) and transcardially perfused with 0.035 l of heparinized (1000 units/l) phosphate buffered saline (PBS, 0.1M, pH 7.4) followed by 0.07 l of 4% paraformaldehyde in phosphate buffer (PB, 0.1M, pH 7.4). The brain was removed and coded. All subsequent procedures were performed by an investigator blind to the procedures performed prior to sacrifice. The brain was immersed in PBS with 20% sucrose and then transferred to 30% sucrose solution for cryoprotection.

Forty-eight hours later, the brain was frozen and cut into 40 μm coronal sections using a sliding microtome (American Optical, US). Each section was placed in one well of a 3-well tray containing PBS, and immunohistochemical procedures were performed immediately. The injection site was further confirmed by visual assessment of the specimen during the sectioning procedure. The remaining tissue was transferred to a cryoprotectant solution and stored at -20°C. For Hcrt quantification during the dark/light condition, all hypothalamic sections from all three wells were combined prior to staining.

### Immunohistochemistry

All immunohistochemical procedures were performed by sequential incubation of free-floating sections. The sections were first incubated in primary antibody for 72 h at 4°C in PBS with 0.25% Triton X-100 (PBST), followed by incubation in the corresponding biotinylated secondary antibody in PBST (Jackson ImmunoResearch, West Grove, PA, USA), standard ABC (Vector Laboratories, Burlingame, CA, USA) and developed with the diaminobenzidine tetrahydrochloride (DAB) method, which consisted of tissue immersion in 0.02% DAB and 0.03% hydrogen peroxide in 10 ml PBS. A common practice in histology laboratories that use the DAB method to develop immunohistochemical procedures, is to periodically examine the tissue sections “under the microscope” during the developing process and remove the sections from the DAB solution once neurons are visualized. However, this approach can add variability to the end result. Therefore, we previously standardized our DAB method and established an 8-minute optimal developing time and used this precise duration in all of our studies. Furthermore all developing solutions were prepared in one container, homogenized and aliquoted in the respective developing wells. All developing procedures were performed with room lights off and the wells containing tissue were additionally wrapped with aluminum foil to protect them from light exposure. Wells were agitated at 55 rpm and all developing solutions were used only once. All procedures were done on coded tissue so that the individual doing the staining and counting did not know the condition of the animals

The following antibodies were used for peptide detection: for Hcrt-1, rabbit anti-Hcrt-1 (H-003-36, Phoenix Pharmaceuticals, USA, 1:2000, Lot # 01108), for MCH, rabbit anti-MCH (H-070-47, Phoenix Pharmaceuticals, USA, 1:20,000, Lot # 01629–3). Identification of histamine and cholinergic neurons was performed by detection of phenotypically specific enzymes histidine decarboxylase (HDC) and choline acetyltransferase (ChAT) using rabbit anti-HDC (EUD 2601, Acris Antibodies, Germany, 1:3000, Lot # ON 2090) or goat anti-ChAT (AB144P, Millipore, USA, 1:2000, Lot # 2464504). All tissue sections from experimental and control animals were stained at the same time and with the same antibody lot.

### Quantification of mRNA; tissue punching, RNA preparation, cDNA synthesis and qPCR

Snap frozen brain samples from mice (8 colchicine, 8 saline) were cut into 200 μm coronal sections using a cryostat (Cryocut 1800, Reichert-Jung, Wien, Austria). All sections corresponding to the tuberal and posterior hypothalamic regions (Paxinos G., 2001) were bilaterally sampled with a 1.0 mm punching needle (Miltex, Inc. York, PA, USA). Each assay tube contained pooled samples from two animals. Brain tissue was homogenized in the presence of QIAzol Lysis Reagent (1000 μl, Qiagne Sciences. Maryland, USA) and chloroform (200 μl, Fisher Scientific. Pittsburgh, PA USA). Isolation of RNA was performed according to the RNeasy Lipid Tissue Mini Kit manufacturer’s protocol (Qiagne Sciences. Maryland, USA) with the addition of RNase-Free DNase kit (Qiagne Sciences. Maryland, USA) to clear potential DNase contamination. The quality and concentration of the RNA was determined with an Eppendorf Bio Spectrometer (Eppendorf. Hauppauge NY, USA). cDNA reactions were conducted immediately, using an iScript cDNA synthesis Kit (Bio-RAD Lab. Inc. Hercules. CA, USA). The Quantitative PCR (qPCR) procedures were performed as previously described [23] [24]. Briefly, the Power SYBR Green PCR kit (20 μl, Life technologies, Warrington UK) with a mixture of sense and antisense primers (2 pmol/μl) was used. GenBank accession number and efficiency for each primer pair are provided in Table 1. Assays were run in a thermocycler (StepOnePlus real-time PCR, Applied Biosystems Grand Island, NY, USA) under the following conditions: 2 min at 50°C and 10 min at 95°C, followed by 40 cycles of 15 s at 95°C and finally 1 min at 60°C. All qPCR assays were conducted in triplicate. Data was acquired and processed automatically using the Applied Biosystems Sequence Detection Software. Specificity of amplification was confirmed by the melting curve and electrophoresis of products on 1.5% Agarose gel. In addition, sterile water (non-template control) and omission of reverse transcriptase (non-RT control) during cDNA synthesis served as negative controls. Amplification efficiency was determined by running qPCR reactions on a dilution series of pooled cDNA from all subjects. The relative expression of the genes of interest was calculated using the corresponding Ct (threshold cycle) values. Samples were normalized to the reference gene Glyceraldehyde-3-Phosphate Dehydrogenase (GAPDH) that is stable between the two groups [25]. The relative amount of target gene in relation to the reference gene was calculated using the 2^(-delta delta Ct) method as described by Livak and Schmittgen (ibid).

**Table 1:** Primers used for qPCR analysis.

Gene	Accession code	Forward primer	Reverse primer	Product length (bp)
GAPDH	NM_008084	GGTGGTGAAGCAGGCATCT	GAAGGTGGAAGAGTGGGAGTT	106
PPHcrt	NM_010410	AACCCATCTTCTATCCTTGTC	CGTCTTTATTGCCATTTACC	83
PPMCH	NM_029971.2	GAACACAGGCTCCAAACA	GCATTCTGAACTCCATTCTC	120

Abbreviations: GAPDH, glyceraldehyde-3-phosphate dehydrogenase; PPHcrt, prepro hypocretin/orexin; PPMCH, prepro melanin concentrating hormone

### Data collection

The number and distribution of Hcrt, HDC, ChAT and MCH cells was assessed using a Nikon Eclipse 80i microscope with three axis motorized stage, video camera, Neurolucida interface and Stereoinvestigator software (MicroBrightField Corp.). Cell counting was performed using the 60x objective and cell size was determined using the Neurolucida Nucleator probe. All counting and cell measurements were performed by a trained histologist, always blind to the experimental condition. In every case, the same individual counted both the experimental and control tissue. Only neurons with an identifiable nucleus were counted.

For quantification of Hcrt neurons in the light/dark phase, both hemispheres in every hypothalamic section were stained and analyzed. In all other experimental groups, quantification of Hcrt, HDC, ChAT and MCH neurons was performed bilaterally on every third section throughout the hypothalamus, and the estimation of the total number of cells was obtained by multiplying the results by the number of wells.

**Fig 1:**
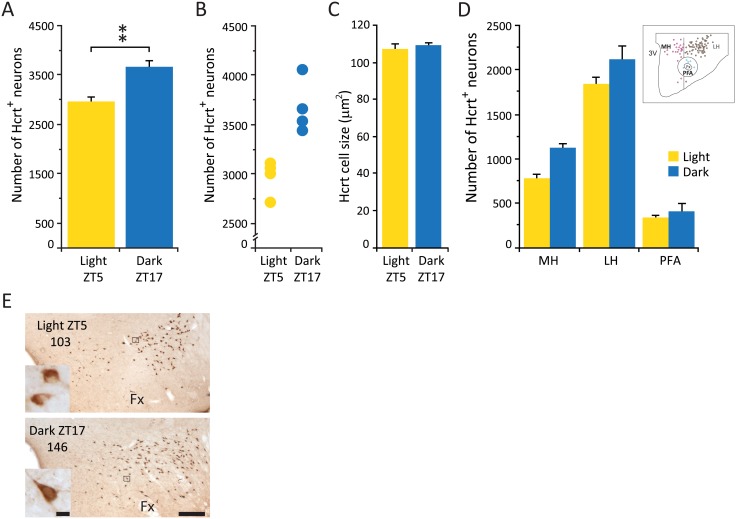
Hcrt neuronal number is increased in dark, but cell size and anatomical distribution are unchanged. (A) Average number of Hcrt cells in the whole hypothalamus. (B) Total cell counts for each experimental subject. (C) Average size of Hcrt neurons. (D) Distribution of Hcrt neurons in different hypothalamic subdivisions. (D) (insert), Diagram of one hypothalamic section of an experimental animal sacrificed during the light phase indicating the anatomical subdivision used, circles represent Hcrt neurons (purple, medial hypothalamus; brown, lateral hypothalamus; blue, perifornical area). (E) Photomicrographs of the same hypothalamic area of an animal sacrificed in the light phase (ZT5, top) and an animal sacrificed in the dark phase (ZT17, bottom). Numbers indicate cell counts on slide. Inserts are higher (x40) magnification photomicrographs of the selected area (black square) for each experimental condition. Calibration bar 250 μm and 10 μm respectively. Fx, fornix; LH, lateral hypothalamus; MH, medial hypothalamus; PFA, perifornical area; 3V third ventricle. ** p = 0.02, Wilcoxon-Mann-Whitney, n = 4 for each group.

In the present study, we divided the hypothalamus into three different anatomical sectors based on previously published work [26]. The perifornical area (PFA) was defined as the region surrounding the fornix (within 140 mm of the perimeter of the structure). The rest of the hypothalamus was further divided into a medial subdivision [medial hypothalamus (MH)], which comprised the area from the medial limit of the fornix to the third ventricle (3V), and a lateral subdivision [lateral hypothalamus (LH)], which extended from the medial boundary of the fornix to the lateral edge of the hypothalamus (see Fig 1D insert).

### Statistical analysis

Non-parametric statistics were used to compare differences among groups. Results were considered statistically significant if p<0.05.

## Results

### Hypocretin/Orexin

Animals that were sacrificed during the dark phase showed a significantly greater number (24%) of Hcrt containing neurons compared to the animals sacrificed during the light phase, (Table 2, Wilcoxon-Mann-Whitney, Z = 2.31, p = 0.02) (Fig 1A). There was no overlap in the individual Hcrt cells counts between the animals in these two different experimental conditions (Fig 1B). All Hcrt neurons were distributed within the hypothalamus as previously described in mice [27] and there was no difference in the total number of hypothalamic sections that contained Hcrt cells (28.8 ± 0.4 and 30 ± 2 for the light and dark respectively). In addition there was no difference in the extension of the Hcrt cell field in the medio-lateral axis (973 ± 16 μm vs 1008 ± 16 μm for the light and dark respectively) as well as in the dorso-ventral axis (620 ± 8 μm vs 649 ± 12 μm for the light and dark respectively) between conditions. This data suggests that the extent of the Hcrt cell population remains unchanged at both time points.

**Table 2:** Number of neurons during the light and dark phase.

	Light phase	Dark phase
Hcrt	2,966 ± 84	3,668 ± 122 **
MCH	5,087 ± 165	4,966 ± 120
ChAt	961 ± 58	995 ± 35
HDC	907 ± 85	1215 ± 43 ^++^

All neuronal counts were determined using the same antibody, lot number and staining parameters.

** Hcrt Dark phase vs. Light phase, p = 0.02;

^++^ HDC Dark phase vs. Light phase, p = 0.02.

There was no significant difference in the overall soma size (106 ± 3 μm^2^ and 109 ± 1.4 μm^2^ for the light and dark respectively) (Fig 1C), and the anatomical parcellation of the hypothalamus (see Methods) did not indicate that the overall increase was restricted to any particular subdivision (Fig 1D). Neuronal morphology appeared indistinguishable between conditions (Fig 1E).

### Histidine decarboxylase

**Fig 2:**
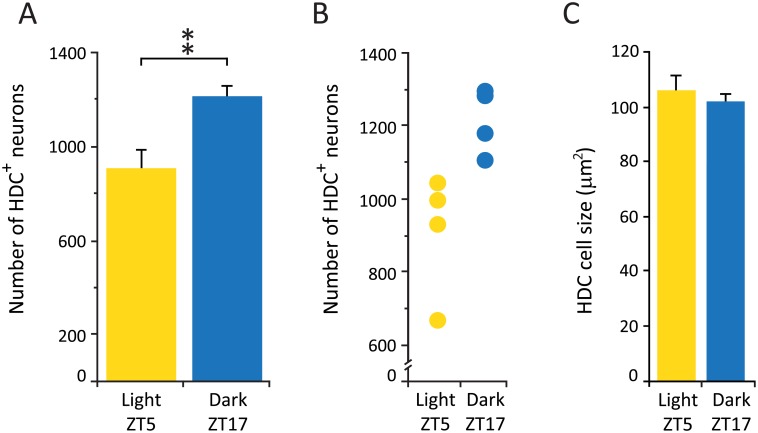
HDC neuronal number is increased in dark, but cell size remains unchanged (like Hcrt cells). (A) Average number of HDC cells in hypothalamic tuberomamillary region. ** p = 0.02, Wilcoxon-Mann-Whitney, n = 4 for each group. (B) Total cell counts for each experimental subject. (C) Average size of HDC neurons.

As seen with the Hcrt cell group, the histaminergic neuronal population also showed a significant increase (33.8%) in the number of neurons detected in animals sacrificed during the dark phase compared to the light phase (Table 2, Wilcoxon-Mann-Whitney, Z = 2.31, p = 0.02) (Fig 2A). In addition, similar to what we observed in the in Hcrt population, there was no overlap in the individual HDC^+^ cell counts between animals in these different experimental conditions (Fig 2B). HDC neurons were located in the tuberomammillary nucleus as previously described in mice [28]. There was no difference in the total number of hypothalamic sections that contained HDC^+^ cells (8.3 ± 0.9 and 9.7 ± 0.6 for the light and dark respectively), and no significant difference in the overall cell size (106 ± 6.7 μm^2^ and 102 ± 3.1 μm^2^ for the dark and light respectively) (Fig 2C). Neuronal morphology appeared indistinguishable between experimental conditions.

### Choline acetyltransferase

**Fig 3:**
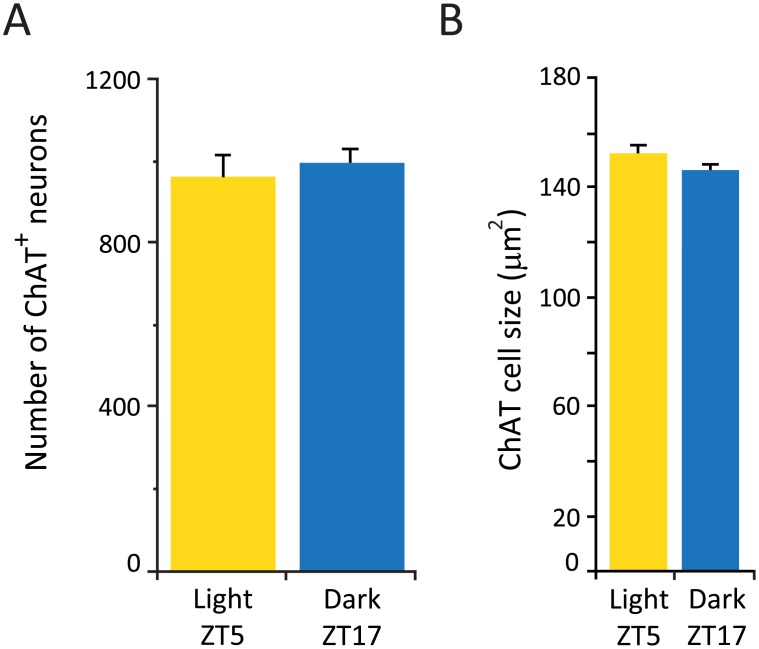
Number and size of ChAT neurons does not vary between the light and dark phase. (A) Average number of ChAT cells in the horizontal diagonal band. n = 4 for each group. (B) Average size of ChAT neurons.

We did not observe a difference in the average number of ChAT^+^ neurons in the caudal sector of the HDB extending from AP: 0.26 mm to 0.02 mm relative to bregma, a forebrain region in which cholinergic cells are concentrated [29] (see below), between the animals sacrificed during the dark and light phase (Fig 3A). In addition, we did not observe a difference in the cell size (153 ± 3.5 μm^2^ and 146 ± 2.1 μm^2^ for the light and dark respectively) (Fig 3B) or in the number of sections that contained cholinergic neurons (10 ± 0.9 and 10 ± 0.9 light and dark respectively).

### Melanin concentrating hormone

**Fig 4:**
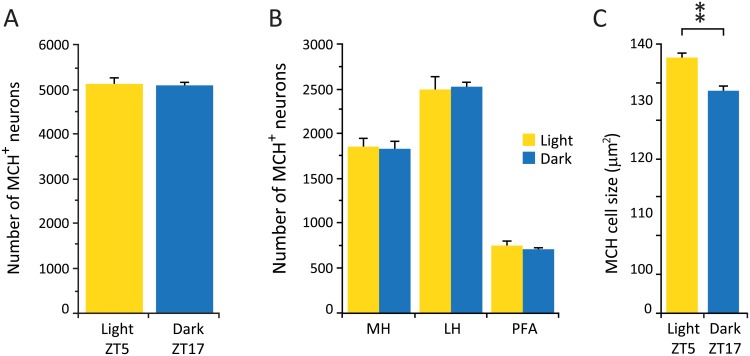
Number of MCH neurons remains unchanged, but cell size is increased in the light. (A) Average number of MCH cells in the light and dark phases. (B) Distribution of MCH neurons in different hypothalamic subdivisions. (C) Average size of MCH neurons. ** p = 0.02, Wilcoxon-Mann-Whitney, n = 4 for each group. LH, lateral hypothalamus; MH, medial hypothalamus; PFA, perifornical area

In contrast to what we observed in the Hcrt and histamine hypothalamic cell groups, there was no significant difference in the average number of MCH neurons between the animals sacrificed during the dark and light phase (Table 2) (Fig 4A). All MCH neurons were distributed within the hypothalamus as previously described in mice [30] and all anatomical subdivisions showed comparable cell counts between experimental conditions (Fig 4B), indicating that there is no shift in the distribution of these neurons between the two time points. However, we observed a significant increase in the average soma size of MCH neurons during the light phase compared to the dark phase (132.8 ± 2.3 μm^2^ and 115.6 ± 2.4 μm^2^ respectively, Wilcoxon-Mann-Whitney, Z = 2.31, p = 0.02) (Fig 4C).

### Colchicine treatment

**Fig 5:**
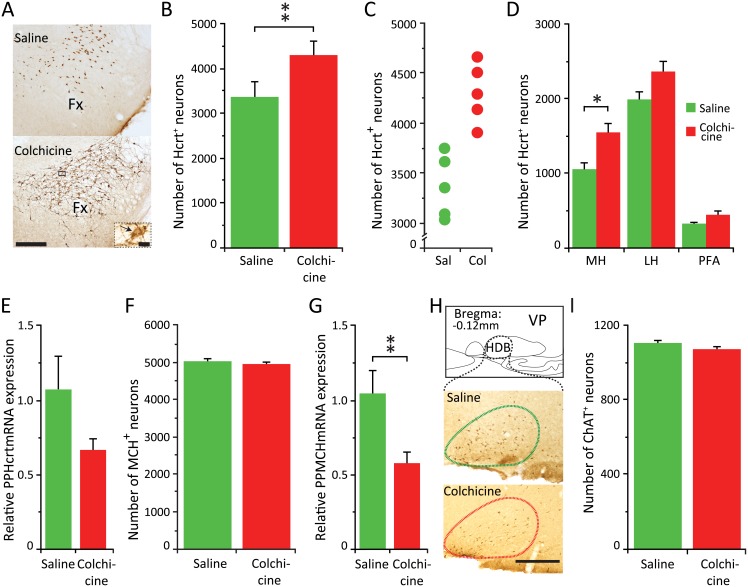
Colchicine further increases the number of Hcrt neurons. **MCH and ChAT neuronal numbers remain unchanged**. (A) Photomicrographs of the same hypothalamic area of an animal treated with saline (top) or colchicine (bottom). Calibration bar 250 μm. Insert corresponds to a higher (x60) magnification of the selected area (black square) of the animal that received colchicine. Black arrow indicates an Hcrt neuron. Calibration bar 10 μm. (B) Total cell counts for each experimental subject. (C) Average number of Hcrt neurons in animals with ICV saline vs. ICV colchicine injections (** p = 0.02, Wilcoxon-Mann-Whitney, n = 5 for each group). (D) Distribution of Hcrt neurons in the three different hypothalamic subdivisions regions. Increase is greatest in the medial hypothalamic region (*p<0.05, Wilcoxon-Mann-Whitney with Bonferroni correction). (E) relative PPHcrt mRNA expression in the hypothalamus. The difference is not significant. (F) Average number of MCH neurons in animals with ICV saline vs. ICV colchicine injections. (G) Relative PPMCH mRNA expression in the hypothalamus (** p = 0.02, Wilcoxon-Mann-Whitney, n = 8 for each group). (H) Diagram of the basal forebrain of the mouse depicting the anatomical localization of the cholinergic cell group analyzed. Lower, photomicrographs of the same basal forebrain area of an animal treated with saline (above) or colchicine (below). In contrast to the increase in the staining of the Hcrt and MCH peptide, staining of ChAT is not increased by colchicine treatment. Calibration bar, 250 μm. (I) Average number of ChAT neurons in animals with ICV saline vs. ICV colchicine injections. LH, lateral hypothalamus; MH, medial hypothalamus; PFA, perifornical area; Fx, fornix; HDB, horizontal diagonal band; VP, ventral pallidum.

The increase in the number of Hcrt cells during the dark phase suggested that during the light phase the amount of Hcrt peptide in the cell bodies of a subgroup of Hcrt neurons was undetectable to our antibody. Therefore we used colchicine, an inhibitor of microtubule polymerization, to accumulate the products of cellular synthesis in the cell body, thus increasing the concentration of Hcrt in all Hcrt producing neurons. As expected, colchicine treated animals showed a more robust Hcrt immunostaining, as illustrated in the photomicrograph in Fig 5A.

In addition, this procedure resulted in an increase in the number of Hcrt neurons detected compared to the saline group (Table 3, Wilcoxon-Mann-Whitney, Z = 2.37, P = 0.02) (Fig 5B). There was no overlap in the individual Hcrt cells counts between the animals in these two different experimental conditions (Fig 5C). To our surprise, the treatment revealed a significantly greater number of Hcrt expressing neurons (17%), than the highest number seen under physiological conditions during the dark phase (Table 3, Wilcoxon-Mann-Whitney, Z = 2.02, p = 0.04) and an overall increase of 44% compared to the light phase condition. However, there was no significant difference in the number of hypothalamic sections containing Hcrt neurons in the colchicine treated animals compared to the dark phase group (35.2 ± 5.6 vs. 30 ± 2 for the colchicine treated and the dark phase respectively). This indicates that all Hcrt expressing neurons detected after the colchicine treatment lay within the previously described extent of the Hcrt neuronal field. The cell number increase was greatest in the medial sector of the hypothalamus compared to the saline treated animals (Wilcoxon-Mann-Whitney, Z = 2.56, p<0.05 with Bonferroni correction) (Fig 5D). Colchicine treatment did not significantly affect the levels of preprohypocretin (PPHcrt) mRNA (PPHcrt mRNA) compared to saline treated animals (Fig 5E).

**Table 3:** Number of neurons after colchicine treatment.

	Saline ICV	Colchicine ICV
Hcrt	3,381 ± 319	4,282 ± 332 ^##^^,^ ^$ $^^,^
MCH	4,871 ± 62	4,936 ± 61
ChAt	1,104 ± 15	1,069 ± 15

All neuronal counts were determined using the same antibody, lot number and staining parameters.

^##^ Hcrt Colchicine ICV vs. Saline ICV, p = 0.02;

^$ $^ Hcrt Colchicine ICV vs. Dark phase, p = 0.04.

In contrast to the situation with Hcrt cells, we did not find a significant increase in the number of MCH cells detected after colchicine treatment, just as we did not see a diurnal difference (Table 3; Fig 5F). We observed a significant reduction in prepro melanin concentrating hormone (PPMCH) mRNA levels (PPMCH mRNA) (Wilcoxon-Mann-Whitney, Z = 2.31, p = 0.02) (Fig 5G) under the colchicine condition.

Colchicine did not affect the number of neurons expressing ChAT (Table 3; Fig 5H and 5I). This is in agreement with previously published data indicating that colchicine has little effect on products that are mainly located in the cell body such as the ChAT enzyme [31]. A similar result would be expected in the histaminergic neuronal group since histaminergic neurons were identified by the presence of HDC enzyme.

## Discussion

### Methodological considerations

The number of immunohistochemically-detected cells in animals with the same genetic background and age can vary as a function of tissue processing [32] [33], the antibody used [14] [15], or the method of collecting data [34] [22]. To minimize experimental sources of variability we processed all subjects’ brains using the same procedures, including antibody lot number. Our work indicates that the level of production in a substantial portion of the Hcrt neurons can fall below immunohistochemical detectability, with non-detection not necessarily an indication of the complete absence of Hcrt.

An alternative method to visualize cells in tissue samples is the detection of specific mRNAs by *in situ* hybridization (ISH). However, the presence of a particular mRNA in a cell does not guarantee the expression of the encoded molecule. In addition, in narcoleptic patients for example, ISH can fail to detect the appropriate PPHcrt mRNA [35] despite the presence of Hcrt neurons (10%), confirmed by immunohistochemistry [36] [13] [37].

### Diurnal rhythm

We report for the first time a significant increase in the number of detectable Hcrt and HDC neurons during the dark phase. This data is compatible with the maximal activity reported for these neuronal groups being during the active phase in rodents [38] [39] [40] [41] [6] [8]. However, it has previously been reported that there is no difference in the number of Hcrt neurons detected by immunohistochemistry across the day-night cycle when assessed in laboratory rodents [40] [42] [43] [44]. There are several explanations for this discrepancy. Martinez et al [42] used just one hemi section per animal for the analysis. The low sampling size would have reduced the statistical power of the results reported. In our study we analyzed every hypothalamic section containing Hcrt immunopositive neurons, quantifying the cell number in both hemispheres for each experimental subject. Estabrooke et al [40], in addition to using a lower sampling size (3 sections per animal), reports that the “…the number of orexin neurons…varied by as much as a factor of two between animals…”. Using the complete counting technique we see a maximum within group variation between subjects of 11.6 to 17.6%. Marston et al [44] conducted their experiments in constant light conditions, and light intensity has been shown to significantly affect the number of neurons expressing Hcrt [45]. Other factors such as species difference [43] might also have contributed to this difference.

The increase in the number of identified Hcrt neurons during the dark phase indicates that a subpopulation of cells augments their Hcrt peptide content at this time point, thus becoming immunohistochemically detectable. This is in accordance with previously published data indicating the highest levels of the Hcrt-1 peptide in the LH, as measured by microdialysis, occurs during the dark phase [46] and may reflect an increase in the synthesis as well as a shift in the anabolism/catabolism of the peptides. The concentration of Hcrt-1 in the pontine region also peaks during the dark phase (ZT18) [47], indicating a higher rate of Hcrt transport to extra hypothalamic targets since, to our knowledge, there are no reports demonstrating Hcrt synthesis in the terminals.

In one scenario, the “non-visible Hcrt cells” belong to a functionally specific subpopulation of Hcrt neurons that increase their Hcrt synthesis only during the dark phase. Alternatively, Hcrt neurons can be “randomly” put "off line" from peptide production during the light phase. Physiological capabilities would be maintained during each phase, but a different population of neurons would be active in light and dark phases.

Analogous to what we observed for the Hcrt system, the increase in the number of histaminergic neurons detected in the dark phase indicates a higher concentration of HDC enzyme within these cells. This could be the result of an augmented synthesis of the enzyme, since the mRNA HDC levels peak during the dark phase [48]. However, other factors like a slower rate of transportation to the terminals, an increased stability of the enzyme or alterations in the rate of degradation may also affect the concentration of the enzyme in the cell body.

The increase in the enzyme content may be partly responsible for the increased levels of this neurotransmitter reported during the dark phase in the anterior and posterior hypothalamus [38] [49]. We cannot determine if the number of HDC cells observed during the dark phase represents the maximum number of histamine neurons, or if there are still more cells that cannot be detected with our immunohistochemical technique. We have previously reported that human narcoleptic patients have a 64% increase in the number of HDC neurons, with an even greater increase reported at the same time by another group [15]. It remains to be determined if a large number of cells capable of producing HDC is present in non-pathological humans and increases their synthesis of HDC as part of the development of narcolepsy, or whether the disease causes a new group of cells to produce HDC.

In contrast with the Hcrt and histamine system, we did not detect a difference in the number of neurons expressing ChAT or MCH at the two time points analyzed, thus the day-night differential is not a universal feature of signaling systems. We observed a significant increase in the average size of the MCH cells during the light phase compared to the dark phase. To our surprise, colchicine treatment did not affect the numbers of MCH neurons. It is possible that MCH neurons have a constantly higher concentration of peptide or the antibody against MCH is more sensitive, therefore all neurons expressing MCH are detected. However we observed a significant decrease in the PPMCH mRNA, possibly the result of a negative feedback loop mechanism elicited by the blockade of transportation of the MCH peptide outside of the cell body.

### Fluctuation in Hcrt cell numbers

A recent study has reported an increase in the number of Hcrt neurons (~20%) following corticosterone treatment [34], indicating that Hcrt production can be increased or induced in a subset of neurons by this steroid. The authors do not make any claims as to the nature of these neurons, thus we speculate that the treatment increases the production of peptides in the "non-visible” population of Hcrt neurons seen under our conditions.

The results of the current study show a naturally occurring increase in the number of Hcrt expressing neurons during the dark phase. However, after blocking microtubule transport we observed an additional 17% increase in the cell counts compared to the highest physiological value we observed (in the dark condition), bringing the number to 44% above the level recorded in the light phase. We cannot rule out the possibility that there is an even greater number of cells capable of producing Hcrt and that the 52 hours of colchicine treatment is not enough to accumulate sufficient peptide to make the cell bodies detectable. However, maintaining the viability of the animals beyond 52 hours is challenging.

We have previously reported that human narcolepsy is characterized by an average loss of 90% of Hcrt neurons. Narcolepsy with cataplexy has not been reported in humans having 15% or more of the average number of Hcrt cells in controls [13] [50]. These results suggest that if it were possible to increase the number of Hcrt cells in human narcoleptics by the ≥17% percentage visible with colchicine treatment, it might be possible to attenuate or reverse narcolepsy with cataplexy. The development of techniques to achieve this and similar changes in other neuronal systems could have a major impact on the treatment of neurological disease.

### Final conclusions

Our study presents evidence that there is a significant fluctuation in the number of detected Hcrt and HDC neurons under physiological conditions. We also show that the total number of potential Hcrt expressing cells is considerably greater than previously thought. Based on the physiological variation and the even greater number of detectable Hcrt neurons seen after colchicine administration, we suggest that these systems may increase or decrease their numbers depending on particular behaviors that accompany the animals’ active phase. Therefore it is not just the activity of the neurons, but the numbers too, implying that target regions innervated by certain neuronal subgroups may receive innervation of a particular transmitter only under specific conditions. These phenomena reveal a new complexity in the dynamics of different identifiable cell groups.

## References

[1] Mizuno T, Endo Y, Arita J, Kimura F (1991) Acetylcholine release in the rat hippocampus as measured by the microdialysis method correlates with motor activity and exhibits a diurnal variation. Neuroscience 44: 607–612. 1754054 10.1016/0306-4522(91)90081-x

[2] Detari L, Rasmusson DD, Semba K (1999) The role of basal forebrain neurons in tonic and phasic activation of the cerebral cortex. Prog Neurobiol 58: 249–277. 10341363 10.1016/s0301-0082(98)00084-7

[3] Lee MG, Hassani OK, Alonso A, Jones BE (2005) Cholinergic basal forebrain neurons burst with theta during waking and paradoxical sleep. J Neurosci 25: 4365–4369. 10.1523/JNEUROSCI.0178-05.2005 15858062 PMC6725118

[4] Lee MG, Hassani OK, Jones BE (2005) Discharge of identified orexin/hypocretin neurons across the sleep-waking cycle. J Neurosci 25: 6716–6720. 10.1523/JNEUROSCI.1887-05.2005 16014733 PMC6725432

[5] Mileykovskiy BY, Kiyashchenko LI, Siegel JM (2005) Behavioral correlates of activity in identified hypocretin/orexin neurons. Neuron 46: 787–798. 10.1016/j.neuron.2005.04.035 15924864 PMC8281334

[6] Shan L, Hofman MA, van Wamelen DJ, Van Someren EJ, Bao AM, et al (2012) Diurnal fluctuation in histidine decarboxylase expression, the rate limiting enzyme for histamine production, and its disorder in neurodegenerative diseases. Sleep 35: 713–715. 10.5665/sleep.1838 22547898 PMC3321431

[7] Passani MB, Panula P, Lin JS (2014) Histamine in the brain. Front Syst Neurosci 8: 64 10.3389/fnsys.2014.00064 24808830 PMC4009418

[8] Blouin AM, Fried I, Wilson CL, Staba RJ, Behnke EJ, et al (2013) Human hypocretin and melanin-concentrating hormone levels are linked to emotion and social interaction. Nat Commun 4: 1547 10.1038/ncomms2461 23462990 PMC3595130

[9] Hassani OK, Lee MG, Jones BE (2009) Melanin-concentrating hormone neurons discharge in a reciprocal manner to orexin neurons across the sleep-wake cycle. Proc Natl Acad Sci U S A 106: 2418–2422. 10.1073/pnas.0811400106 19188611 PMC2650171

[10] Luppi PH, Clement O, Fort P (2013) Paradoxical (REM) sleep genesis by the brainstem is under hypothalamic control. Curr Opin Neurobiol 23: 786–792. 10.1016/j.conb.2013.02.006 23490549

[11] Pelluru D, Konadhode R, Shiromani PJ (2013) MCH neurons are the primary sleep-promoting group. Sleep 36: 1779–1781. 10.5665/sleep.3196 24293750 PMC3825425

[12] Haas HL, Sergeeva OA, Selbach O (2008) Histamine in the nervous system. Physiol Rev 88: 1183–1241. 10.1152/physrev.00043.2007 18626069

[13] Thannickal TC, Moore RY, Nienhuis R, Ramanathan L, Gulyani S, et al (2000) Reduced number of hypocretin neurons in human narcolepsy. Neuron 27: 469–474. 11055430 10.1016/s0896-6273(00)00058-1 PMC8760623

[14] John J, Thannickal TC, McGregor R, Ramanathan L, Ohtsu H, et al (2013) Greatly increased numbers of histamine cells in human narcolepsy with cataplexy. Ann Neurol. 10.1002/ana.23968 PMC8211429 23821583

[15] Valko PO, Gavrilov YV, Yamamoto M, Reddy H, Haybaeck J, et al (2013) Increase of histaminergic tuberomammillary neurons in narcolepsy. Ann Neurol. 10.1002/ana.24019 24006291

[16] Nitz D, Andersen A, Fahringer H, Nienhuis R, Mignot E, et al (1995) Altered distribution of cholinergic cells in the narcoleptic dog. Neuroreport 6: 1521–1524. 7579139 10.1097/00001756-199507310-00014 PMC9051663

[17] Tafti M, Nishino S, Liao W, Dement WC, Mignot E (1997) Mesopontine organization of cholinergic and catecholaminergic cell groups in the normal and narcoleptic dog. J Comp Neurol 379: 185–197. 9050784 10.1002/(sici)1096-9861(19970310)379:2<185::aid-cne2>3.0.co;2-#

[18] Mikrouli E, Wortwein G, Soylu R, Mathe AA, Petersen A (2011) Increased numbers of orexin/hypocretin neurons in a genetic rat depression model. Neuropeptides 45: 401–406. 10.1016/j.npep.2011.07.010 21871662

[19] Muschamp JW, Dominguez JM, Sato SM, Shen RY, Hull EM (2007) A role for hypocretin (orexin) in male sexual behavior. J Neurosci 27: 2837–2845. 10.1523/JNEUROSCI.4121-06.2007 17360905 PMC6672590

[20] Perekrest SV, Abramova TV, Novikova NS, Loskutov YV, Rogers VJ, et al (2008) Changes in immunoreactivity of orexin-A-positive neurons after intravenous lipopolysaccharide injection. Med Sci Monit 14: BR127–133. 18591911

[21] Santollo J, Eckel LA (2013) Oestradiol decreases melanin-concentrating hormone (MCH) and MCH receptor expression in the hypothalamus of female rats. J Neuroendocrinol 25: 570–579. 10.1111/jne.12032 23414264 PMC3668853

[22] Palomba M, Seke-Etet PF, Laperchia C, Tiberio L, Xu YZ, et al (2015) Alterations of orexinergic and melanin-concentrating hormone neurons in experimental sleeping sickness. Neuroscience 290: 185–195. 10.1016/j.neuroscience.2014.12.066 25595977

[23] Shan L, Bossers K, Luchetti S, Balesar R, Lethbridge N, et al (2012) Alterations in the histaminergic system in the substantia nigra and striatum of Parkinson's patients: a postmortem study. Neurobiol Aging 33: 1488 e1481–1413. 10.1016/j.neurobiolaging.2011.10.016 22118942

[24] Shan L, Bossers K, Unmehopa U, Bao AM, Swaab DF (2012) Alterations in the histaminergic system in Alzheimer's disease: a postmortem study. Neurobiol Aging 33: 2585–2598. 10.1016/j.neurobiolaging.2011.12.026 22284987

[25] Livak KJ, Schmittgen TD (2001) Analysis of relative gene expression data using real-time quantitative PCR and the 2(-Delta Delta C(T)) Method. Methods 25: 402–408. 10.1006/meth.2001.1262 11846609

[26] McGregor R, Wu MF, Barber G, Ramanathan L, Siegel JM (2011) Highly specific role of hypocretin (orexin) neurons: differential activation as a function of diurnal phase, operant reinforcement versus operant avoidance and light level. J Neurosci 31: 15455–15467. 10.1523/JNEUROSCI.4017-11.2011 22031892 PMC3230273

[27] Peyron C, Tighe DK, van den Pol AN, de Lecea L, Heller HC, et al (1998) Neurons containing hypocretin (orexin) project to multiple neuronal systems. J Neurosci 18: 9996–10015. 9822755 10.1523/JNEUROSCI.18-23-09996.1998 PMC6793310

[28] Parmentier R, Ohtsu H, Djebbara-Hannas Z, Valatx JL, Watanabe T, et al (2002) Anatomical, physiological, and pharmacological characteristics of histidine decarboxylase knock-out mice: evidence for the role of brain histamine in behavioral and sleep-wake control. J Neurosci 22: 7695–7711. 12196593 10.1523/JNEUROSCI.22-17-07695.2002 PMC6757981

[29] Berger-Sweeney J, Stearns NA, Murg SL, Floerke-Nashner LR, Lappi DA, et al (2001) Selective immunolesions of cholinergic neurons in mice: effects on neuroanatomy, neurochemistry, and behavior. J Neurosci 21: 8164–8173. 11588189 10.1523/JNEUROSCI.21-20-08164.2001 PMC6763842

[30] Bittencourt JC, Presse F, Arias C, Peto C, Vaughan J, et al (1992) The melanin-concentrating hormone system of the rat brain: an immuno- and hybridization histochemical characterization. J Comp Neurol 319: 218–245. 10.1002/cne.903190204 1522246

[31] Ichikawa T, Ajiki K, Matsuura J, Misawa H (1997) Localization of two cholinergic markers, choline acetyltransferase and vesicular acetylcholine transporter in the central nervous system of the rat: in situ hybridization histochemistry and immunohistochemistry. J Chem Neuroanat 13: 23–39. 9271193 10.1016/s0891-0618(97)00021-5

[32] Obukuro K, Nobunaga M, Takigawa M, Morioka H, Hisatsune A, et al (2013) Nitric oxide mediates selective degeneration of hypothalamic orexin neurons through dysfunction of protein disulfide isomerase. J Neurosci 33: 12557–12568. 10.1523/JNEUROSCI.0595-13.2013 23904594 PMC6618541

[33] Yamaguchi K, Futatsuki T, Ushikai J, Kuroki C, Minami T, et al (2015) Intermittent but not sustained hypoxia activates orexin-containing neurons in mice. Respir Physiol Neurobiol 206: 11–14. 10.1016/j.resp.2014.11.003 25462014

[34] Jalewa J, Wong-Lin K, McGinnity TM, Prasad G, Holscher C (2014) Increased number of orexin/hypocretin neurons with high and prolonged external stress-induced depression. Behav Brain Res 272: 196–204. 10.1016/j.bbr.2014.05.030 24867335

[35] Peyron C, Faraco J, Rogers W, Ripley B, Overeem S, et al (2000) A mutation in a case of early onset narcolepsy and a generalized absence of hypocretin peptides in human narcoleptic brains. Nat Med 6: 991–997. 10.1038/79690 10973318

[36] Lin L, Faraco J, Li R, Kadotani H, Rogers W, et al (1999) The sleep disorder canine narcolepsy is caused by a mutation in the hypocretin (orexin) receptor 2 gene. Cell 98: 365–376. 10458611 10.1016/s0092-8674(00)81965-0

[37] Crocker A, Espana RA, Papadopoulou M, Saper CB, Faraco J, et al (2005) Concomitant loss of dynorphin, NARP, and orexin in narcolepsy. Neurology 65: 1184–1188. 10.1212/01.wnl.0000168173.71940.ab 16247044 PMC2254145

[38] Mochizuki T, Yamatodani A, Okakura K, Horii A, Inagaki N, et al (1992) Circadian rhythm of histamine release from the hypothalamus of freely moving rats. Physiol Behav 51: 391–394. 1313592 10.1016/0031-9384(92)90157-w

[39] Fujiki N, Yoshida Y, Ripley B, Honda K, Mignot E, et al (2001) Changes in CSF hypocretin-1 (orexin A) levels in rats across 24 hours and in response to food deprivation. Neuroreport 12: 993–997. 11303775 10.1097/00001756-200104170-00026

[40] Estabrooke IV, McCarthy MT, Ko E, Chou TC, Chemelli RM, et al (2001) Fos expression in orexin neurons varies with behavioral state. J Neurosci 21: 1656–1662. 11222656 10.1523/JNEUROSCI.21-05-01656.2001 PMC6762959

[41] Takahashi K, Lin JS, Sakai K (2006) Neuronal activity of histaminergic tuberomammillary neurons during wake-sleep states in the mouse. J Neurosci 26: 10292–10298. 10.1523/JNEUROSCI.2341-06.2006 17021184 PMC6674640

[42] Martinez GS, Smale L, Nunez AA (2002) Diurnal and nocturnal rodents show rhythms in orexinergic neurons. Brain Res 955: 1–7. 12419515 10.1016/s0006-8993(02)03264-x

[43] Kodama T, Usui S, Honda Y, Kimura M (2005) High Fos expression during the active phase in orexin neurons of a diurnal rodent, Tamias sibiricus barberi. Peptides 26: 631–638. 10.1016/j.peptides.2004.11.016 15752578

[44] Marston OJ, Williams RH, Canal MM, Samuels RE, Upton N, et al (2008) Circadian and dark-pulse activation of orexin/hypocretin neurons. Mol Brain 1: 19 10.1186/1756-6606-1-19 19055781 PMC2632999

[45] Deats SP, Adidharma W, Lonstein JS, Yan L (2014) Attenuated orexinergic signaling underlies depression-like responses induced by daytime light deficiency. Neuroscience 272: 252–260. 10.1016/j.neuroscience.2014.04.069 24813431 PMC4090246

[46] Yoshida Y, Fujiki N, Nakajima T, Ripley B, Matsumura H, et al (2001) Fluctuation of extracellular hypocretin-1 (orexin A) levels in the rat in relation to the light-dark cycle and sleep-wake activities. Eur J Neurosci 14: 1075–1081. 11683899 10.1046/j.0953-816x.2001.01725.x

[47] Taheri S, Sunter D, Dakin C, Moyes S, Seal L, et al (2000) Diurnal variation in orexin A immunoreactivity and prepro-orexin mRNA in the rat central nervous system. Neurosci Lett 279: 109–112. 10674633 10.1016/s0304-3940(99)00955-6

[48] Yu X, Zecharia A, Zhang Z, Yang Q, Yustos R, et al (2014) Circadian factor BMAL1 in histaminergic neurons regulates sleep architecture. Curr Biol 24: 2838–2844. 10.1016/j.cub.2014.10.019 25454592 PMC4252164

[49] Rozov SV, Zant JC, Karlstedt K, Porkka-Heiskanen T, Panula P (2014) Periodic properties of the histaminergic system of the mouse brain. Eur J Neurosci 39: 218–228. 10.1111/ejn.12397 24438489

[50] Thannickal TC, Nienhuis R, Siegel JM (2009) Localized loss of hypocretin (orexin) cells in narcolepsy without cataplexy. Sleep 32: 993–998. 19725250 10.1093/sleep/32.8.993 PMC2717206

